# Sleep disturbances and quality of life in Sub-Saharan African migraineurs

**DOI:** 10.1186/s10194-015-0504-x

**Published:** 2015-03-04

**Authors:** Isabel Morgan, Francisco Eguia, Bizu Gelaye, B Lee Peterlin, Mahlet G Tadesse, Seblewengel Lemma, Yemane Berhane, Michelle A Williams

**Affiliations:** Department of Epidemiology, Harvard T.H. Chan School of Public Health Multidisciplinary International Research Training Program, 677 Huntington Ave, K505F, Boston, 02115 MA USA; Department of Neurology, Johns Hopkins University School of Medicine, Baltimore, MD USA; Department of Mathematics & Statistics, Georgetown University, Washington, DC USA; Addis Continental Institute of Public Health, Addis Ababa, Ethiopia

**Keywords:** Migraine, Sleep quality, Quality of life, Ethiopia

## Abstract

**Background:**

Although in the past decade occidental countries have increasingly recognized the personal and societal burden of migraine, it remains poorly understood in Africa. No study has evaluated the impact of sleep disturbances and the quality of life (QOL) in sub-Saharan Africans with migraine.

**Methods:**

This was a cross-sectional study evaluating adults, ≥ 18 years of age, attending outpatient clinics in Ethiopia. Standardized questionnaires were utilized to collect demographic, headache, sleep, lifestyle, and QOL characteristics in all participants. Migraine classification was based on International Classification of Headache Disorders (ICHD)-II criteria. The Pittsburgh Sleep Quality Index (PSQI) and the World Health Organization Quality of Life (WHOQOL-BREF) questionnaires were utilized to assess sleep quality and QOL characteristics, respectively. Multivariable logistic regression models were fit to estimate adjusted odds ratio (OR) and 95% confidence intervals (95% CI).

**Results:**

Of 1,060 participants, 145 (14%) met ICHD-II criteria for migraine. Approximately three-fifth of the study participants (60.5%) were found to have poor sleep quality. After adjustments, migraineurs had over a two-fold increased odds (OR = 2.24, 95% CI 1.49-3.38) of overall poor sleep quality (PSQI global score >5) as compared with non-migraineurs. Compared with non-migraineurs, migraineurs were also more likely to experience short sleep duration (≤7 hours) (OR = 2.07, 95% CI 1.43-3.00), long sleep latency (≥30 min) (OR = 1.97, 95% CI 1.36-2.85), daytime dysfunction due to sleepiness (OR = 1.51, 95% CI 1.12-2.02), and poor sleep efficiency (<85%) (OR = 1.93, 95% CI 1.31-2.88). Similar to occidental countries, Ethiopian migraineurs reported a reduced QOL as compared to non-migraineurs. Specifically Ethiopian migraineurs were more likely to experience poor physical (OR = 1.56, 95% CI 1.08-2.25) and psychological health (OR = 1.75, 95% CI 1.20-2.56), as well as poor social relationships (OR = 1.56, 95% CI 1.08-2.25), and living environments (OR = 1.41, 95% CI 0.97-2.05) as compared to those without migraine.

**Conclusion:**

Similar to occidental countries, migraine is highly prevalent among Ethiopians and is associated with poor sleep quality and a lower QOL. These findings support the need for physicians and policy makers to take action to improve the quality of headache care and access to treatment in Ethiopia.

## Background

Migraine is a common neurological disorder characterized by localized, intense, throbbing or pulsing sensations in the head, which is often accompanied by nausea, vomiting, photophobia and phonophobia [1]. Globally migraine is responsible for roughly 3% of disability making it the 8^th^ most burdensome disease [2]. Research on migraine and primary headache disorders is relatively expansive in volume, but not in geographical scope, with the majority of research coming from higher income countries, and disproportionately less knowledge coming from lower income countries. In sub-Saharan Africa, where only five countries have contributed to primary headache disorder research, nine articles have been published in the span of almost ten years (1997–2006) [3]. In the past five years, there have been few studies investigating the burden of migraine [4-12] in accordance with International Classification of Headache Disorder (ICHD) criteria. A recent study conducted among Ethiopian adults found a migraine prevalence of 9.8% comparable to what is reported in occidental countries (10-15%) [5].

Sleep disorders, affecting more than 45% of the world’s population, have emerged as important global public health problems [13,14]. An emerging body of epidemiologic evidence supports that sleep disorders are highly prevalent among sub-Saharan Africans [15,16]; and are important risk factors for headache attacks [17,18] as well as other adverse health outcomes [19-24]. Specifically, studies have shown that sleep disorders are associated with daytime sleepiness (assessed by direct observation and/or based on self-report), and reduced perception of overall quality of life (QOL) [25]. However the health consequences of sleep disorders have not been thoroughly evaluated among sub-Saharan Africans. Additionally, disrupted sleep-wake patterns have been shown to predispose individuals to headache attacks and increase the risk of chronification [17,18,26]. Further, improvement in sleep quality has long been described as beneficial for migraineurs [27].

Studies have also shown that migraineurs are more likely to have a lower QOL as compared with non-migraineurs [28,29]. Measures of QOL provide a quantitative assessment of an individual’s social relationships, living environment, physical and psychological health status [30]. QOL measures also provide the aggregate burden imposed by specific health conditions such as migraine headache and sleep disturbances [31]. To the best of our knowledge, no study has comprehensively evaluated the burden of sleep disturbances and QOL among sub-Saharan African migraineurs. Given the increased understanding of migraine prevalence among sub-Saharan Africans, it is crucial to gain a better understanding of migraine and its relationship with sleep disturbances and QOL in this population. Information from this and similar studies may provide the evidence needed for developing preventive and therapeutic strategies designed to mitigate the burden of both conditions in these and other understudied populations.

## Methods

### Design and participants

This study consisted of 1,060 participants who attended the Saint Paul General Hospital outpatient facility (M/F: 423/637; mean age 35.7 ± 12.1 years) in Addis Ababa, Ethiopia. The hospital provides all basic services across various medical departments including: pediatrics, internal medicine, gynecology, neurology, general surgery, psychiatry, ophthalmology, and emergency medicine. For this study only patients evaluated in the internal medicine, general surgery and gynecological outpatient departments were eligible for inclusion. Data collection was conducted between July and December, 2011. Study personnel included research nurses with public health training. Prior to the start of the study, research nurses participated in a four- day training session that included training modules on the contents of the questionnaire, ethical conduct of human subjects research, and appropriate data collection techniques. Structured in-person interviews were used to collect information regarding demographics, headache characteristics, sleep disturbances, QOL, and behavioral and lifestyle characteristics. The interview included a structured migraine assessment questionnaire (adapted from previously validated structured interviews although not specifically validated in our study population) [32,33]. The questionnaire was first written in English and then translated by linguistic experts into Amharic and was translated back into English. Using a recruitment script, research nurses approached patients and invited them for participation. Approximately 85% of individuals who were invited to participate in the study elected to do so. All study participants provided informed consent and all research protocols were approved by the Institutional Review Boards of Addis Continental Institute of Public Health, Addis Ababa, Ethiopia and the Human Subjects Division at the University of Washington, Seattle, USA.

### Variable specification

Migraine was classified based on the ICHD-II criteria [34]. Migraine (ICHD-II category 1.1 or 1.2) was defined by at least five lifetime headache attacks (criterion A) lasting 4–72 hours (criterion B), with at least two of the qualifying pain characteristics [unilateral location (criterion C1), pulsating quality (criterion C2), moderate or severe pain intensity (criterion C3), aggravation by routine physical exertion (criterion C4)]; at least one of the associated symptoms [nausea and/or vomiting (criterion D1), photo/phonophobia (criterion D2)]; and not attributable to another central nervous system disorder or head trauma (according to subject self-report) (criterion E).

### Pittsburgh Sleep Quality Index (PSQI)

Sleep quality was assessed using the Pittsburgh Sleep Quality Index (PSQI) [35]. The PSQI is a 19-item self-reported, validated questionnaire that evaluates sleep quality within the past month. The PSQI consists of seven sleep components related to sleep habits including duration of sleep, sleep disturbance, sleep latency, habitual sleep efficiency, use of sleep medicine, daytime dysfunction and overall sleep quality. The sleep components yield a score ranging from 0 to 3, with three indicating the greatest dysfunction [35]. The sleep component scores are summed to yield a total score ranging from 0 to 21, with higher total or global scores indicating poor sleep quality. Based on prior literature [35], participants with a global score greater than 5 were classified as poor sleepers. Those with a score of 5 or less were classified as good sleepers.

For sleep quality component subscales, a dichotomous variable of optimal and suboptimal sleep quality was utilized. Specific categories included long sleep latency (≥30 minutes vs. < 30 minutes); poor sleep efficiency (<85% vs. ≥85%); days dysfunction due to sleep (< once a week vs. ≥ once per week); and sleep medication use during the past month (< once per week vs. ≥ once per week). Sleep duration was assessed using the PSQI questionnaire that queried participants how many hours of actual sleep the participants got at night during the previous month. In accordance with the original scale, the following groupings were used to define sleep duration: <5 hours, 5.1-5.9 hours, 6–6.9 hours, and ≥ 7 hours. Those in the lowest three groups of sleep duration (<7 hours) were classified as short duration sleepers.

### World Health Organization quality of life questionnaire

The WHO QOL questionnaire (WHO-QOL) [36] is a cross-cultural assessment tool that captures an individual’s perception of their position in life in the context of culture and the value systems in which they live and in relation to their goals, expectations, standards and concerns [36]. In the current study the abbreviated version of WHO-QOL, the WHOQOL-BREF, was utilized and includes 26 items across four domains. The physical domain consists of questions about daily activities, dependence on medication and treatment, energy and exhaustion, mobility, pain and discomfort, sleep and rest, and capacity to work. The psychological domain consists of questions about positive and negative feelings, self-esteem, body image and external image, personal beliefs, and attention. The social relationships domain consists of questions about relationships with others, social support, and sex life. The environmental domain of the scale consists of questions about the home environment, physical security and safety, financial resources, availability of health services, leisure activities, physical environment, and transportation. The overall percentile score for each domain ranges from 0% (very poor) to 100% (very good).

### Covariates

Questions were also included regarding behavioral risk factors such as tobacco, alcohol, and khat consumption. Khat is an evergreen plant with amphetamine-like effects commonly used as a mild stimulant for social recreation and to improve work performance in Ethiopia [37,38]. Participants were classified according to their alcohol consumption habits: nondrinker (<1 alcoholic beverage a week), moderate (1–21 alcoholic beverages a week), and high to excessive consumption (>21 alcoholic beverages a week) according to the WHO classification [39]. Other variables were categorized as follows: age (years), sex (male, female), education (≤ high school, technical school, ≥ bachelor’s degree), smoking history (never, former, current), and current Khat consumption (yes, no). Participants were also asked the following question about their self-reported health status derived from the WHO questionnaire [40]: “Would you say your health in general is excellent, very good, good, fair, or poor?” and were classified into those who reported fair or poor health and those who reported excellent, very good, or good health. Self-reported health status is an important construct that provides vital information about how one perceives and report’s one’s own well-being [41].

### Statistical analysis

All analyses were performed using SPSS Version 22.0, (IBM SPSS Version 22, Chicago, IL,). Demographic and lifestyle characteristics of study participants were assessed using means (± standard deviation) for continuous variables and counts and percentages for categorical variables. For skewed variables median [interquartile range] were provided. Differences in categorical variables were evaluated using Chi-square test or Fisher’s exact test as appropriate. For continuous variables with normal distributions, Student’s *t*-tests were used to evaluate differences in mean values by migraine status. Unadjusted, age and sex-adjusted, and multivariable models, were fit to estimate odds ratios (ORs) and 95% confidence intervals (CI) for the associations of migraine and sleep quality parameters (i.e., sleep duration, latency, efficiency and overall sleep quality, daytime dysfunction and sleep medicine use in the past month). Multivariable-adjusted logistic regression models controlled for age, sex, body mass index, alcohol consumption, and smoking status. Confounding variables were considered *a priori* for documented associations of migraine [42] with sleep disorders and QOL. Independent sample *t*-tests were used to compare differences in mean values for QOL scores. To determine statistically significant correlates of migraine among the QOL domains, we performed similar unadjusted and multivariate-adjusted logistic regression models as described above. Given the lack of standard cut-offs for the domains in the WHOQOL-BREF, we used quartiles to define the groups and used the lowest quartile as the reference group in the analyses. All reported *p-*values are two-sided and defined as significant at the 5% level.

## Results

### Migraine prevalence

Table 1 reports the demographic characteristics of the sample. Of 1,060 participants, 145 (14%) met ICHD-II criteria for migraine. A majority of the sample were women (60.1%), married (51.3%), and with less than a 12^th^ grade education (78.4%). Approximately 4% reported current smoking status, while 56.7% reported no alcohol consumption in the past year. Current khat consumption was reported by 20.6% of participants. Poor physical health and mental health were self-reported by 43.8% and 34.1% of the sample, respectively. Overall, migraine prevalence was greater among women (16.8%) than men (8.9%), p < 0.001.

**Table 1:** **Characteristics of the study population (N = 1,060)**

	**N = 1,060**	
**Characteristic**	**n**	**%**
Mean age (years)*	35.7 ± 12.1	
Sex		
Women	637	60.1
Men	423	39.9
Marital status		
Married	542	51.3
Never married	335	31.7
Other	180	17.0
Education (years)		
≤ Primary (1–6)	474	44.7
Secondary (7–12)	357	33.7
College graduate	229	21.6
Smoking status		
Never	913	86.1
Former	104	9.8
Current	43	4.1
Alcohol consumption past year		
Non-drinker	601	56.7
< once a month	357	33.7
≥1 day a week	102	9.6
Khat chewing		
None	783	73.9
Former	59	5.6
Current	218	20.6
Body mass index (kg/m^2^)		
<18.5	174	16.5
18.5-24.9	629	59.7
24.9-29.9	184	17.5
≥30	67	6.4
Self-reported physical health		
Excellent/very good/good	596	56.2
Poor/fair	464	43.8
Self-reported mental health		
Excellent/very good/good	699	65.9
Poor/fair	361	34.1

*Mean ± standard deviation (SD).

### Sleep habits/patterns and migraine

Table 2 summarizes sleep quality parameters (PSQI sleep component scales). 43.3% of subjects reported short sleep duration <7 hours, while 47.9% of subjects reported long sleep latency (>30 minutes). Overall, 599 (56.5%) and 234 (22%) subjects reported poor sleep efficiency (<85%) and daytime dysfunction more than once a week, respectively. Some 60.2% of participants were classified as having suboptimal/poor overall sleep quality. Overall, there was no statistically significant difference between men and women with regards to sleep duration, sleep latency, daytime dysfunction, sleep efficiency, sleep medicine use or overall sleep quality (data not shown). Participants’ characteristics, according to migraine status, are summarized in Table 2. Migraineurs were more likely to report short sleep duration, long sleep latency, daytime dysfunction due to sleepiness, poor sleep efficiency, and sleep medication use during the past month as compared with non-migraineurs. Overall, poor sleep quality (PSQI global score > 5) was more common among migraineurs as compared with non-migraineurs (75.9% vs. 58.0%).

**Table 2:** **Pittsburgh sleep quality index components according to migraine**

	**All N = 1,060**	**Migraine**		
		**Yes** **N = 145**	**No** **N = 915**	
Characteristic	n (%)	n (%)	n (%)	P-value*
Sleep duration (hours)				
<5	106 (10.0)	17 (11.7)	89 (9.7)	0.002
5-5.9	126 (11.9)	27 (18.6)	99 (10.8)	
6-6.9	227 (21.4)	39 (26.9)	188 (20.6)	
≥7	601 (56.7)	62 (42.8)	539 (58.9)	
Sleep latency (minutes)				
≤15	293 (27.6)	32 (22.1)	261 (28.5)	<0.001
16-30	259 (24.4)	23 (15.9)	236 (25.8)	
31-60	277 (26.1)	41 (28.3)	236 (25.8)	
>60	231 (21.8)	49 (33.8)	182 (19.9)	
Day dysfunction due to sleep				
Never	443 (41.8)	44 (30.3)	399 (43.6)	0.001
< once a week	383 (36.1)	57 (39.3)	326 (35.6)	
1-2 times per week	189 (17.8)	31 (21.4)	158 (17.3)	
≥3 times per week	45 (4.2)	13 (8.9)	32 (3.5)	
Sleep efficiency (%)				
≥85	461 (43.5)	45 (31.0)	416 (45.5)	0.012
75-84	140 (13.2)	23 (15.9)	117 (12.8)	
65-74	81 (7.6)	15 (10.3)	66 (7.2)	
<65	378 (35.7)	62 (42.8)	316 (34.5)	
Sleep medicine during past month				
Never	1,024 (96.6)	136 (93.8)	888 (97.0)	0.039
< once a week	7 (0.7)	2 (1.4)	5 (0.6)	
1-2 times per week	13 (1.2)	5 (3.4)	8 (0.9)	
≥3 times per week	16 (1.5)	2 (1.4)	14 (1.5)	
Overall sleep quality				
Good	419 (39.8)	35 (24.1)	384 (42.0)	<0.001
Poor	641 (60.2)	110 (75.9)	531 (58.0)	

*Chi-square test or Fisher’s test (when the sample size in a cell < 5).

The odds ratios (OR) for migraine across the PSQI sleep quality parameters are shown in Table 3. In the multivariable-adjusted model, migraineurs were more likely than non-migraineurs to report short sleep duration (<7 hours) (OR = 2.05, 95% CI 1.42-2.97), long sleep latency (>30 min) (OR = 1.97, 95% CI 1.36-2.85), daytime dysfunction due to sleepiness (OR = 1.50, 95% CI 1.00-2.25), and poor sleep efficiency (<85%) (OR = 1.94, 95% CI 1.32-2.85). Migraineurs also had a 1.73-fold increased odds of taking sleep medications (95% CI 0.68-4.39) than non-migraineurs although statistical significance was not achieved. Finally, migraineurs had a 2.26-fold increased odds (OR = 2.26, 95% CI 1.50-3.39) of overall poor sleep quality (PSQI global score >5) as compared with non-migraineurs.

**Table 3:** **Odds Ratios (OR) and 95% Confidence Intervals (CI) of sleep quality in relation migraine status**

**Sleep quality parameters**	**Unadjusted**	**Age and sex adjusted OR (95% CI)**	**Multivariable adjusted** ^*^ **OR (95% CI)**
Poor sleep quality			
No	1.00 (Reference)	1.00 (Reference)	1.00 (Reference)
Yes	2.27 (1.52-3.39)	2.31 (1.59-3.46)	2.26 (1.50-3.39)
Short sleep duration (<7 hours)			
No	1.00 (Reference)	1.00 (Reference)	1.00 (Reference)
Yes	1.91 (1.34-2.73)	2.01 (1.40-2.89)	2.05 (1.42-2.97)
Long sleep latency (>30 min)			
No	1.00 (Reference)	1.00 (Reference)	1.00 (Reference)
Yes	1.94 (1.36-2.79)	1.90 (1.32-2.73)	1.97 (1.36-2.85)
Day dysfunction due to sleep			
No	1.00 (Reference)	1.00 (Reference)	1.00 (Reference)
Yes	1.66 (1.12-2.45)	1.60 (1.08-2.37)	1.50 (1.00-2.25)
Poor sleep efficiency (<85%)			
No	1.00 (Reference)	1.00 (Reference)	1.00 (Reference)
Yes	1.85 (1.27-2.69)	1.92 (1.31-2.82)	1.94 (1.32-2.85)
Sleep medicine use			
No	1.00 (Reference)	1.00 (Reference)	1.00 (Reference)
Yes	2.06 (0.86-4.91)	1.68 (0.66-4.24)	1.73 (0.68-4.39)

*Adjusted for age, sex, body mass index, alcohol consumption, smoking status.

### Quality of life and migraine

As shown in Table 4, when compared with non-migraineurs, migraineurs scored significantly lower in all four WHO-QOL domains measured. In the physical domain, mean scores for migraineurs were significantly lower (−3.7 points; p value = 0.003) than non-migraineurs. In the psychological domain, migraineurs had a mean score of 56.2 as compared with 49.0 among non-migraineurs (p < 0.001). The mean score differential between migraineurs and non-migraineurs were 6.0 (p = 0.004) and 4.0 (p = 0.005) in the social and environmental domains, respectively.

**Table 4:** **Mean WHO quality of life scores according to migraine**

**Quality of life assessed by domain**	**Migraine**				**P-value**
	**No (N = 915)**		**Yes (N = 145)**		
	**Mean score**	**SD**	**Mean score**	**SD**	
Physical	51.6	13.7	47.9	12.2	0.003
Psychological	56.2	18.0	49.0	18.3	<0.001
Social relationships	63.8	22.9	57.8	24.3	0.004
Environmental	42.3	16.0	38.3	14.4	0.005

Table 5 shows the association between migraine and the WHO-QOL domains. In the multivariable-adjusted model, compared with non-migraineurs, migraineurs were more likely to experience poor physical health (OR = 1.56, 95% CI 1.08-2.25), psychological health (OR = 1.75, 95% CI 1.20-2.56), social relationships (OR = 1.56, 95% CI 1.08-2.25), and living environment (OR = 1.41, 95% CI 0.97-2.05).

**Table 5:** **Odds Ratios (OR) and 95% Confidence Intervals (CI) of quality of life in relation to migraine**

**WHOQOL-BREF**	**Unadjusted**	**Age and sex adjusted OR (95% CI)**	**Multivariable adjusted** ^*^ **OR (95% CI)**
Poor physical health			
No	1.00 (Reference)	1.00 (Reference)	1.00 (Reference)
Yes	1.55 (1.08-2.22)	1.56 (1.09-2.24)	1.56 (1.08-2.25)
Poor psychological health			
No	1.00 (Reference)	1.00 (Reference)	1.00 (Reference)
Yes	1.80 (1.24-2.59)	1.76 (1.21-2.54)	1.75 (1.20-2.56)
Poor social relationships			
No	1.00 (Reference)	1.00 (Reference)	1.00 (Reference)
Yes	1.49 (1.04-2.13)	1.57 (1.09-2.25)	1.56 (1.08-2.25)
Poor living environment			
No	1.00 (Reference)	1.00 (Reference)	1.00 (Reference)
Yes	1.42 (0.98-2.04)	1.38 (0.95-2.00)	1.41 (0.97-2.05)

*Adjusted for age, sex, body mass index, alcohol consumption, smoking status; WHOQOL-BREF: World Health Organization quality of life questionnaire abbreviated version of.

## Discussion

In this study of sub-Saharan Africans approximately 14% of the study sample was found to have migraine while more than 60% of participants were classified as having poor sleep quality. Migraineurs, as compared with non-migraineurs, were more likely to report experiences of short sleep duration, long sleep latency, daytime dysfunction due to sleepiness, poor sleep efficiency and overall poor sleep quality, independent of age, sex, BMI, alcohol consumption and smoking status. Furthermore, migraineurs were more likely to report having significantly impaired QOL as compared with non-migraineurs.

A majority of study participants (60.5%) were classified as having poor sleep quality. This finding is consistent with some [43,44] although not all [45,46] previous epidemiologic studies. Nuhu *et al.* [43], in their study of primary care patients attending an outpatient clinic in Nigeria, reported that 68.7% of participants had poor sleep quality [43]. Similarly Zhu *et al.* [44], in a study of Chinese migraineurs recruited from a tertiary hospital headache clinic, reported poor sleep quality among 61.6% of participants. Investigations of sleep quality in other populations, however, have reported lower prevalence rates. For instance Minowa *et al.* [46], using the PSQI, reported that 30-45% of 4,868 Japanese white-collar workers suffered from poor sleep quality, while Hoefelmann *et al.* [45] reported that 21% of 47,304 Brazilian industrial workers had poor sleep quality. At least in part, these differences across studies may be attributable to differences in geographic locations, operational definitions of sleep quality and characteristics of the study populations.

Over the past three decades, the importance of proper, sustained sleep has garnered increasing interest within the public health community [47]. Both migraine and sleep disturbances overlap in symptom profiles with other chronic disorders. Poor sleep quality and habits have been linked to loss of work productivity [46,48], impaired QOL [49,50], increased risk for cardiovascular diseases [49], and psychiatric morbidities [18,51-54]. The co-morbidities with poor sleep quality and other sleep disturbances are well documented, primarily in high-income countries [54-56]. Our study expands the literature by providing evidence that these associations with poor sleep and reduced quality of sleep in migraineurs extends to lower income countries.. Given the under-recognition and under-treatment of migraine and sleep disorders in sub-Saharan Africa and other low and middle income regions of the world, these findings will have important clinical and global public health implications. Understanding their comorbidity may provide opportunities to enhance the diagnoses and treatment of these prevalent disorders.

We found migraineurs had increased odds of experiencing poor QOL in all domains compared with non-migraineurs. This finding is in agreement with prior literature. For instance, in their population-based study of Russian adults, Ayzenberg *et al*. [28] found that migraineurs were more likely to have poor WHOQOL scores compared with non-migraineurs (27.1 ± 4.9 vs. 30.5 ± 4.5; p < 0.05). Similarly Arslantas *et al*. [57] in Turkey reported that migraineurs had significantly impaired health related QOL across all domains of the 36-item short-form (SF-36) except for the general health perception, social functioning, and role-emotional domains. In sub-Saharan Africa where migraine diagnosis and treatment remain very low, the impairments in QOL might cause distress and frustration for the migraineurs and their families. The results of the present study and those of others [28] further support the importance of early identification and that proper treatment of migraine can significantly improve QOL.

Migraine episodes are largely commonplace with regular onset patterns –either at night or in the early morning following bouts of disturbed sleep- suggesting that sleep patterns may influence or exacerbate migraine symptoms [18,58]. Several plausible and compelling biological mechanisms have been proposed underlying the observed migraine–sleep disorder association. Some investigators suspect an underlying connection is controlled by the hypothalamus-pituitary- adrenal axis (HPA), in which the hypothalamus may not respond efficiently to physiological triggers instigated by sleep disturbances [18,58]. Migraineurs with aura often report several common premonitory symptoms including yawning and daytime sleepiness, which are hypothesized to be mediated by dopamine and influenced by the HPA axis. Conversely, Engstrom *et al.* [59] hypothesize that arousability is mediated by the autonomic nervous system and possibly alters pain thresholds among migraineurs. Further, given that migraine is primarily understood as a neurovascular condition, hypotheses related to increased sensitivity to vasodilation may explain the link between migraine and sleep disturbances [60,61].

Several limitations of this work must be taken into consideration. First, we used a cross-sectional study design, which restricts the assertion of temporality between migraine, sleep disorders and QOL. Future prospective studies in which sleep quality habits and QOL are recorded in migraineurs and non-migraineurs would allow for better understanding of the directionality of reported association. Second, our study population consists of patients who attended the Saint Paul General Hospital outpatient facility, thereby limiting the generalizability of our study findings to a broader general population. Third, migraine diagnosis was made based upon information collected using a well-established structured questionnaire [32,33]. Use of structured interviews remains the most feasible method of data collection for large scale epidemiologic studies. Further, the potential of recall bias must be considered as participants were asked to report sleep habits over the past month to address PSQI questions. Lastly, subjective sleep quality and habits were measured using the self-administered PSQI assessment tool. While the PSQI assess subjective sleep quality, it is a leading assessment tool, tested for internal consistency [35] and used to ascertain sleep quality parameters in diverse global populations [62,63].

Results from our study indicate that similar to occidental countries, migraine is highly prevalent in Ethiopia; and that those with migraine have increased odds of experiencing poor sleep quality and lower QOL. Migraine is a disabling neurological disorder with substantial impact on personal health, work productivity, QOL and co-morbidity with other chronic diseases [2]. The findings of our study and those of others from sub-Saharan Africa re-iterate that migraine is not a mere inconvenience as it is often portrayed [4-12] and support the need for physicians and policy makers to take action to improve the quality of headache care and access to treatment in sub-Saharan Africa.
